# The “speed limit” for macromolecular crystal growth

**DOI:** 10.1002/pro.3491

**Published:** 2018-10-16

**Authors:** Renee J. Arias, Jens T. Kaiser, Douglas C. Rees

**Affiliations:** ^1^ Division of Chemistry and Chemical Engineering California Institute of Technology Pasadena California, 91125; ^2^ Howard Hughes Medical Institute California Institute of Technology Pasadena California, 91125

**Keywords:** protein crystallization, enzyme mechanism, protein nanocrystals, micro‐electron diffraction

## Abstract

A simple “diffusion‐to‐capture” model is used to estimate the upper limit to the growth rate of macromolecular crystals under conditions when the rate limiting process is the mass transfer of sample from solution to the crystal. Under diffusion‐limited crystal growth conditions, this model predicts that the cross‐sectional area of a crystal will increase linearly with time; this prediction is validated by monitoring the growth rate of lysozyme crystals. A consequence of this analysis is that when crystal growth is diffusion‐limited, micron‐sized crystals can be produced in ~1 s, which would be compatible with the turnover time of many enzymes. Consequently, the ability to record diffraction patterns from sub‐micron sized crystals by X‐ray Free Electron Lasers and micro‐electron diffraction technologies opens the possibility of trapping intermediate enzyme states by crystallization.

## Introduction

Innovations in structural biology are expanding the capabilities for studying macromolecular structure and function through diffraction experiments on sub‐micron sized crystals utilizing X‐ray Free Electron Lasers (XFEL) and micro‐electron diffraction (MicroED).^1^, ^2^, ^3^, ^4^ Among these new capabilities are evolving opportunities for the crystallographic characterization of enzymatic reaction intermediates. With traditional diffraction sources, the study of reaction intermediates through time‐resolved crystallography has been limited to those exceptional cases where either suitable photoactivatable substrates are available or where the enzyme kinetics are sufficiently slow that the reaction may be triggered by diffusion of substrates into the crystal.^5^ The timescale for the diffusion of substrates into crystals depends on the accessibility of a substrate to the active site in the crystal, and diffusion times for various enzymes and substrates have been reported that range from multiple seconds to hours.^6^, ^7^, ^8^, ^9^ As the typical kcat for enzyme catalysis with physiological substrates is ~10 s^−1^ (Ref. 10), it is, therefore, not possible to characterize intermediate states for many enzymes by substrate diffusion into crystals, which has motivated a number of approaches for preparing stable complexes using combinations of mutations, inhibitors, and solution conditions. Furthermore, for some reactions, such as nitrogenase, it is not possible to simply diffuse in substrates to trigger the reaction, as even though the physiological substrate is the diatomic N_2_, the enzyme mechanism involves multiple proteins and large conformational changes that are incompatible with the crystal lattice. As an alternative approach to ligand diffusion, we consider the possibility of trapping intermediates by rapid crystallization from an enzyme solution undergoing turnover.

The motivation for this study arose from our previous work on the nitrogenase MoFe‐protein where we crystallized the CO‐inhibited form of the protein from a sample generated under turnover conditions in a heterogeneous mixture.^11^ Utilizing a seeding strategy, suitable crystals were obtained in ~4 h that enabled data collection on a third‐generation synchrotron source to a sufficiently high resolution (1.5 Å) to identify CO bound to an electron dense metallocluster. While this technology was successful for trapping an inhibited form of the MoFe‐protein, it would be inadequate to trap intermediates generated during the reduction of N_2_ or other substrates since the turnover time of the enzyme (~1 s) is much shorter than the time for crystallization.

Crystallization is a complex process and extensive experimental and theoretical models have been developed, taking into account key parameters in crystallization conditions including protein and precipitant concentrations, pH, temperature, salt, etc.^12^, ^13^ The kinetics of crystal growth reflect the rates of the underlying processes including nucleation, diffusion of sample from the surrounding solution to the crystal surface, and the surface kinetics by which molecules add to the growing surface.^12^, ^14^, ^15^, ^16^ The “speed limit” for crystal growth is ultimately set by the diffusion rate, which will occur when all other processes (nucleation and surface kinetics) are faster. To estimate this limit, we used the “diffusion‐to‐capture” model to evaluate the timescale for diffusion‐controlled crystal growth. We then experimentally determined the growth rates of lysozyme crystals to assess the relevance of the theoretical model for capturing the basic kinetics of crystal growth.

## Diffusion‐to‐Capture Model

Crystal growth involves the incorporation of material diffusing to the surface from the surrounding solution. The upper limit to crystal growth can be estimated using the “diffusion‐to‐capture” model based on the steady‐state solution to Fick's second law.^17^ Modeling a crystal as a sphere, the growth rate under diffusion‐limited conditions can be evaluated from the diffusion current describing the rate at which mass, *M*, is transferred to the crystal from solution at a bulk concentration of C_0_:

(1) $$\frac{\mathrm{dM}}{\mathrm{dt}}=4\pi {\mathrm{rDC}}_{o}\;$$

where *D* is the diffusion coefficient and r is radius; in SI units, *M*, *D*, *C*_0_, and *r* have units of kg, m^2^ s^−1^, kg m^−3^ (= mg ml^−1^), and m, respectively. The mass of protein in a spherical crystal will depend on *r*, the crystal density *ρ* (kg m^−3^), and the volume fraction f of protein in the crystal:

(2) $$M=\frac{4}{3}\pi r^{3}\mathrm{f\rho}$$

The time derivatives of the crystal mass and radius are related through the following equation:

(3) $$\frac{\mathrm{dM}}{\mathrm{dt}}=4\pi r^{2}\mathrm{f\rho}\frac{\mathrm{dr}}{\mathrm{dt}}$$

The growth rate d*r*/d*t* may be evaluated by relating Eqs. (1) and (3):

(4) $$\frac{\mathrm{dr}}{\mathrm{dt}}=\frac{1}{4\pi r^{2}\mathrm{f\rho}}\frac{\mathrm{dM}}{\mathrm{dt}}=\frac{4\pi {\mathrm{rDC}}_{o}}{4\pi r^{2}\mathrm{f\rho}}=\frac{{\mathrm{DC}}_{o}}{r\;\mathrm{f\rho}}\;$$

Under diffusion‐limited conditions, the growth rate of a spherical crystal is not constant, but varies inversely with the radius of the crystal.^14^

The time required for a spherical crystal to grow from radius 0 to R may be obtained by integrating Eq. (4) to give

(5) $$\begin{array}{l} \int_{0}^{t}\mathrm{dt}=\int_{0}^{R}\frac{\mathrm{rf\rho}}{{\mathrm{DC}}_{o}}\mathrm{dr}\\ t\left(R\right)=\frac{\mathrm{f\rho}}{2{\mathrm{DC}}_{o}}R^{2} \end{array}$$

This analysis predicts that the time for a spherical crystal to reach a radius *R* increases as *R* ^2^ or equivalently, *t*(*R*) is proportional to the cross‐sectional area *A* (= *π*R^2^). For the crystallization of a “typical” protein, *D* ~ 10^−10^ m^2^ s^−1^, *ρ* ~ 1300 kg m^−3^, *f* ~ 0.5, and *C*_0_ ~ 10 kg m^−3^ (= 10 mg ml^−1^), so that

(6) $$\begin{array}{l} t\left(R\right)=\frac{\mathrm{f\rho}}{2\pi {\mathrm{DC}}_{o}}\left(\pi R^{2}\right)=\frac{\mathrm{f\rho}}{2\pi {\mathrm{DC}}_{o}}A\\ =3\times 10^{11}\;R^{2}=1\times 10^{11}\;A\;\left(\text{in}\;m^{2}\right)\;\\ =0.3\;R^{2}=0.1\;A\;\left(\text{in}\;\mu m^{2}\right) \end{array}$$

This analysis indicates that under conditions when diffusion is rate limiting, crystals of radius 1, 10, and 100 μm could grow in ~0.3 s, 0.5 min, and 1 h, respectively. Equivalently, the cross‐sectional area under these conditions is predicted to increase by 10 μm^2^ s^−1^. While the linear growth rate d*r*/d*t* decreases as the crystal size increases, for crystals of radius ~50 μm (plausibly a typical size), the diffusion limited growth rate (Eq. (4)) is estimated as 0.03 μm s^−1^; for comparison, the growth rates for different crystal faces of lysozyme have been reported as ~ 0.01–0.08 μm s^−1^, measured from a single face of the growing crystal.^12^, ^18^, ^19^

## Results and Discussion

**Figure 1:**
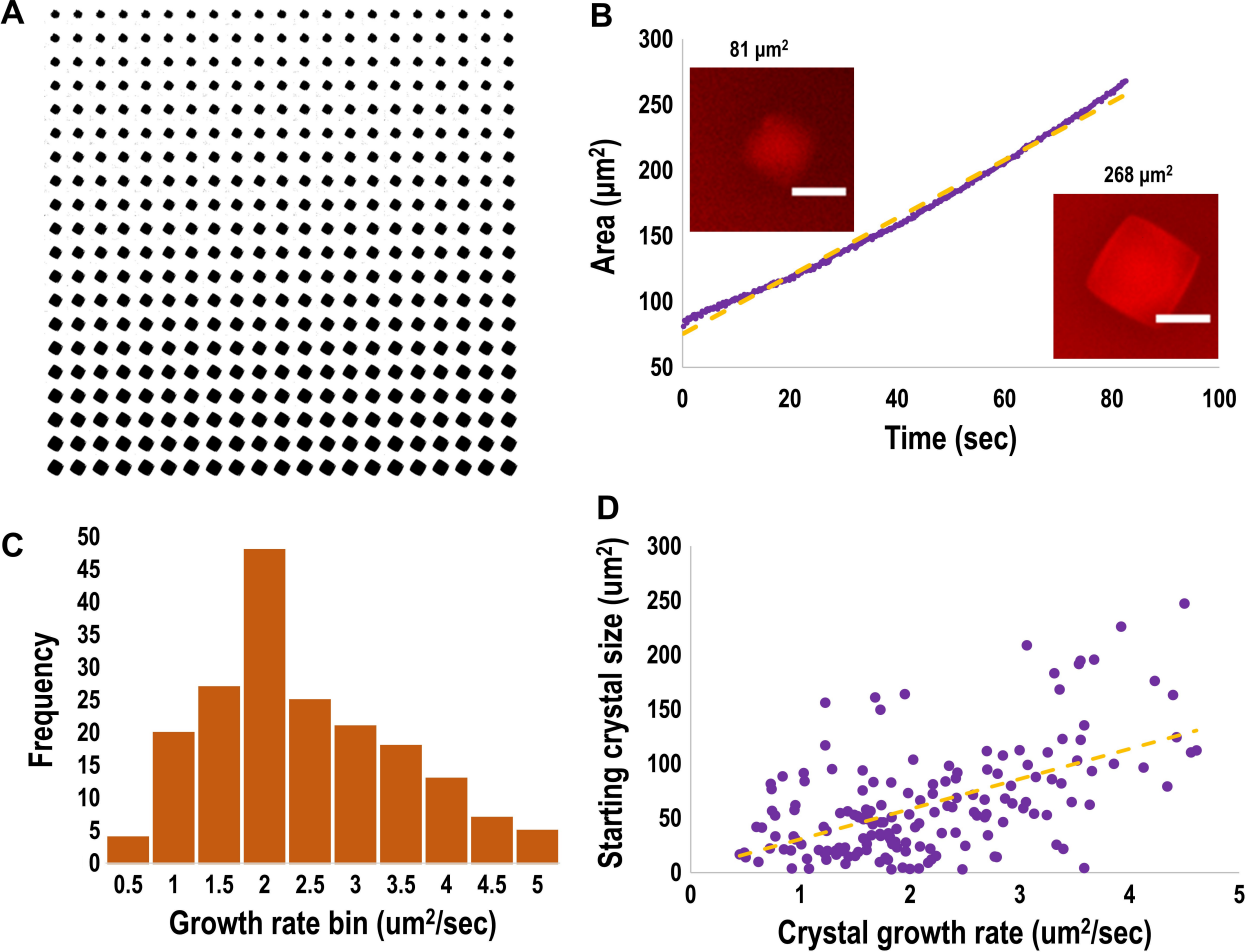
(A) Binary montage of the growth of a CR‐labeled lysozyme crystal generated from a sequence of images recorded with a fluorescence microscope. The elapsed time for this sequence is 82 s. (B) Plot of the increase in cross‐sectional area (μm^2^) over time (s) for the growing crystal depicted in the montage. Image analysis was performed with the ImageJ processing program.^26^ The orange trend line shows a linear fit of 2.2 μm^2^ s^−1^, with *R* ^2^ = 0.99. Inset, *left*: still image cropped to the selected crystal at beginning of the time‐lapse video. Inset, *right*: still image of the same crystal at the end of the time‐lapse video. Scale bar is 10 μm. (C) Growth rates of 168 crystals separated into bins of 0.5 μm^2^ s^−1^. (D) Growth rates correlated to starting crystal size in μm^2^. The orange trendline was fit to the linear equation 27.7 *x* + 3.0, with Pearson's coefficient = 0.552.

To experimentally validate the theoretical diffusion‐limited growth rate of protein crystals, we devised a method to monitor crystal growth rates. We chose lysozyme for this study, as it crystallizes readily and is often used in crystal growth studies.^18^, ^20^ The crystal growth kinetics were monitored using lysozyme labeled with the fluorescent dye carboxyrhodamine‐red (CR) to permit visualization of crystal growth in capillaries by fluorescence microscopy (Fig. 1). The low labeling level employed (estimated at <0.5%) did not appear to interfere with the formation of tetragonal lysozyme crystals, as confirmed by a 2.0 Å resolution crystal structure (data not shown).

With this experimental system, the growth of a single CR‐labeled lysozyme crystal was recorded in a series of snapshots over 82 s [Fig. 1(a)]. The cross‐sectional area of each image was then evaluated and plotted as a function of time [Fig. 1(b)]; the area was observed to increase in an approximately linear fashion at a rate of ~2.2 μm^2^ s^−1^, although an upward curvature to the trend line is also evident. We proceeded to collect video recordings of 168 growing crystals, which were then analyzed to determine the corresponding growth rates (Fig. 1). Collectively, this analysis established the average cross‐sectional area growth rate of lysozyme crystals was 2.1 (±0.9) μm^2^ s^−1^, with an observed maximum of 4.6 μm^2^ s^−1^. The diffusion‐to‐capture model (Eq. (6)) predicts a diffusion‐limited growth rate of ~10 μm^2^ s^−1^, which is within a factor of 2 of the growth rates observed here. The diffusion‐limited model also predicts that the increase in cross‐sectional area with time should be independent of the magnitude of the cross‐sectional area of each crystal. The observation of a slight positive correlation between crystal size and growth rate [Fig. 1(d)] suggests that diffusion may not be completely rate limiting for crystal growth.

The ability to grow micron‐sized crystals in ~1 s under diffusion‐limited crystal growth conditions opens the possibility of using crystallization to trap intermediates under turnover conditions for structural analysis. X‐ray crystallography can be preferable to cryo‐EM single particle analysis for particular uses, such as experiments where absorption edges and anomalous diffraction data are collected, as is the case for many metalloproteins.^4^, ^11^ Further, crystallography is still the most consistent method to obtain high resolution structures (below 2 Å). While this analysis supports a “speed limit” for crystal growth compatible with formation of micron‐sized crystals under turnover conditions, it also highlights the challenge of devising experimental systems where crystal growth is diffusion‐limited on this time scale. Nucleation of crystal growth represents a major obstacle to this goal, as nucleation is a multi‐phase process with complex kinetics evolving over minutes to hours (see Ref. 21). The use of seeding techniques provides an approach to overcome the nucleation barrier; one possible approach would be development of rapid crystallization systems based on microfluidics systems.^22^, ^23^ Injection ports on the microfluidic device would be used to introduce the enzyme turnover system and crystallization components (including seed crystals), while an outlet port could be adapted for droplet collection at the desired size and/or timepoint. The crystals could then be analyzed by direct injection into an XFEL source or by rapid freezing on electron microscopy grids for subsequent microED analysis. Although undoubtedly quite demanding technically, rapid crystallization methods represent a possible approach for surmounting the challenging problem of structurally characterizing transient intermediates at high resolution.

## Methods

### *Protein labeling and quantification*

Lysozyme from chicken egg white (Sigma lyophilized powder, protein ≥90%, ≥40,000 units/mg protein) was prepared in 50 m*M* sodium acetate (pH 4.5) at 12.5 mg/mL concentration. The protein was labeled with carboxyrhodamine‐red (CR) using the Trace Fluorescence Labeling kit from Molecular Dimensions (MD1–73). The labeling procedure was repeated approximately 5 times to concentrate the labeled product to a concentration of ~60 mg/mL, which was done using Amicon centrifugal filters with MWCO 3000. Protein quantification was done by UV–VIS absorption spectroscopy, using an extinction coefficient of ε = 36 mM^−1^ for lysozyme at 280 nm (Ref. 24).

### *Crystallization*

Lysozyme microcrystals were formed in a 16‐channel Microlytic Crystal Former (Anatrace, CF‐O‐20) with ~60 mg/mL labeled lysozyme in 50 m*M* sodium acetate, pH 4.5, with less than 0.5% labeling efficiency according to the kit and previous estimations.^25^ The crystallization solution was 15% PEG 6000, 3.4 M NaCl, and 1 M sodium acetate (pH 4.5). Crystals were formed by pipetting 0.5 μL of labeled lysozyme into one end of the crystal former channel, and pipetting 0.5 μL of crystallization solution into the other end. The use of the Microlytic Crystal Formers alleviated microscope focusing issues encountered with larger crystal wells.

### *Crystal growth rates*

Crystal growth rates were observed using a Leica light microscope and Hamamatsu camera (C8484‐05G02), set to excite CR at ~530 nm. Time lapse videos were collected using the software Slidebook to capture growing crystals as soon as the crystallization solution was pipetted into the Microlytic Crystal Former. Videos were collected at 10× magnification.

### *Analysis*

Two hundred ninety‐eight crystal growth videos were collected. Of the 298 videos, 168 were readily analyzable by ImageJ software^26^ (no background interference, no frame gaps, no interfering crystals, etc.). The image scale was calibrated using the dimensions of the crystal former (100 μm × 150 μm × 10 mm) in ImageJ. The average frame time was recorded in the Slidebook software, and applied to every frame in a selected sequence. Each crystal in the 10× video was cropped into individual timelapses and analyzed separately. The image was converted to a black and white image using the Binary function in ImageJ. From the binary image, each particle was analyzed for cross‐sectional area over time using the Analyze Particles function in ImageJ. Each crystal growth rate was plotted in Excel using the average frame rate recorded from SlideBook, and the cross‐sectional area obtained from ImageJ. Growth rates were then reported collectively from 168 crystals.
